# Genome-Wide Association Study Heterogeneous Cohort Homogenization via Subject Weight Knock-Down

**DOI:** 10.1371/journal.pone.0048653

**Published:** 2012-10-29

**Authors:** André X. C. N. Valente, Joseph Zischkau, Joo Heon Shin, Yuan Gao, Abhijit Sarkar

**Affiliations:** 1 Systems Biology Group, Biocant – Biotechnology Innovation Center, Cantanhede, Portugal; 2 Center for Neuroscience and Cell Biology, University of Coimbra, Coimbra, Portugal; 3 Center for the Study of Biocomplexity, Virginia Commonwealth University, Richmond, Virginia, United States of America; 4 Physics Department and Vitreous State Laboratory, Catholic University of America, Washington DC, United States of America; 5 Lieber Institute for Brain Development, Johns Hopkins Medical Campus, Baltimore, Maryland, United States of America; Queen's University Belfast, United Kingdom

## Abstract

Population structure can be a source of both false-positive and false-negative findings in a genome-wide association study. This article proposes an approach that helps to reduce the false-positives. It consists of homogenizing the diseased/healthy phenotype ratio across the cohort, by decreasing the statistical weight of selected individuals. After homogenization, the cohort is statistically handled as if originating from a single well-mixed population. The method was applied to homogenize a Parkinson's disease genome-wide association study cohort.

## Introduction

The genome-wide association study (GWAS) is nowadays routinely used to discover genetic susceptibilities to disease [1], [2], [3]. In these studies, a critical step is the handling of population structure in the analyzed cohort [4], [5]. If not correctly accounted for, population structure can result in both false-positive and false-negative phenotype-genotype associations. We briefly review some of the main approaches currently utilized to identify and correct for the presence of population structure in a cohort [6], [7], [8]. Genomic control is a computationally fast and easy to implement method [9], [10], [11]. It prescribes a reduction by a factor λ in the cohort size utilized in test statistical calculations. This compensates for statistical significance inflation due to the presence of population structure. The method assumes that only the few strongest statistical associations reflect genuine phenotype-genotype associations and thus it estimates the λ factor based on the remaining bulk of the test statistics distribution. Dadd et al. [12] discuss refinements and variations on the genomic control approach. An example is the use of multiple rather than a single adjustment factor [13]. A different approach is to first capture ancestry by changing the cohort data to the principal component coordinates of a space defined by a set of markers assumed to be independent of the trait under analysis [14], [15]. The first few principal components can then be utilized as regression covariates in the subsequent association analysis [16], [17]. Other population structure correction approaches based on the calculated principal components have also been proposed [18], [19], [20], [21]. As an alternative to principal component analysis, population structure can also be captured by the multidimensional-scaling (MDS) statistical technique [20], [22], [23]. Li et al. [24], report a method that combines MDS with a phylogeny constructed from SNP genotypes. Spectral graph theory provides yet a different way to capture genetic ancestry. Two implementations of this approach are Spectral-GEM [25] and LAPSTRUCT [26]. Structured association methods first assign to individuals probabilities of membership in given subpopulations [27], [28]. Association testing is then conditional on these subpopulation membership probabilities [29]. STRUCTURE/STRAT [30] and ADMIXMAP [31] are standard software packages that implement this method. Structured association approaches tend to be computationally intensive, but the GWAS analysis package Plink [32] includes a simplified, efficient version of structured association. Finally, linear mixed models [33], [34] have been also successfully applied to address population structure. Wu reports a performance comparison of some of the above approaches [35].

To assist in reducing the specific case of false-positives, this article suggests the additional avenue of homogenizing the ratio between the two GWAS phenotypes (e.g., diseased and healthy) throughout the cohort. The homogenization is performed within a principal component coordinates space and is accomplished by knocking-down the statistical weight of selected individuals. After homogenization, the cohort is statistically handled as if originating from a single well-mixed population. First, under the idealization of exactly two distinct populations, we recall the biases introduced by population structure in a GWAS. We then present our homogenization approach for the practical case where the cohort population structure has a continuous character. The method is described alongside its application to the homogenization of a Parkinson's disease GWAS cohort [36]. Finally, the method is tested using simulated, synthetic data.

## Analysis

### Two populations case

**Figure 1:**
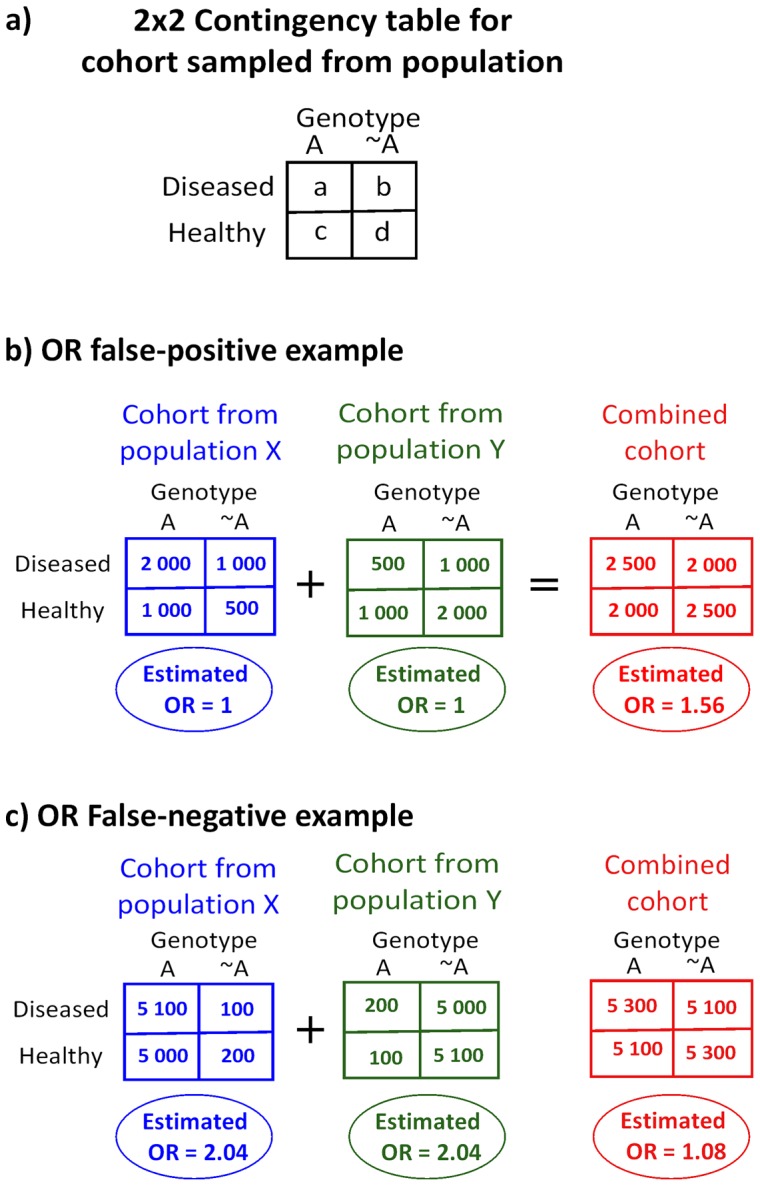
Odds ratio estimation biases introduced by population structure. **a**) The 2×2 contingency table associated with a cohort sampled from the population. Merging cohorts from distinct populations can produce both false-positive and false-negative assessments of the odds ratio (OR). Differences in the sampling process invalidate a straightforward combination of cohorts from distinct populations. **b**) False-positive example: In both population X and population Y, the OR = 1. **c**) False-negative example: In both population X and population Y, the OR≈2.

Consider a population of individuals classified into two genotypes (A and ∼A) and likewise classified into two phenotypes (diseased and healthy). The genotype-phenotype population odds ratio (OR) [37] quantifies the degree of correlation between genotype and phenotype intrinsic to the population. A cohort sampled from the population provides an estimate of the OR. One of the four degrees of freedom (DOFs) of the sampled cohort's 2×2 contingency table (Figure 1-a) can thus be assigned to the OR estimate. Call it the *OR DOF*. The remaining three DOFs then reflect how the cohort was sampled from the population. These three *sampling DOFs* may be expressed as:

- *p*_1_ - number of patients sampled,
- *p*_2_ - number of healthy subjects sampled and
- *p*_3_ - sampling partiality towards A subjects versus towards ∼A subjects.

Any selection for the three sampling DOFs is valid, as they are independent of the OR DOF. Still, correctness of the OR estimate requires every patient and healthy subject to be sampled under the same partiality value *p*_3_. However, population differences in the relative prevalence of A and ∼A subjects are conducive to different *p*_3_ sampling partialities. Therefore casually combining samples from different populations is problematic, as illustrated by the following two examples. Consider two populations X and Y, where in both the OR is unity (Figure 1-b). Let population X have a preponderance of genotype A and population Y have a preponderance of genotype ∼A, thus inducing different sampling partialities *p*_3_. Additionally, regarding DOFs *p*_1_ and *p*_2_, suppose in population X mostly patients were sampled (i.e., *p*_1_ >> *p*_2_) while in population Y mostly healthy controls were sampled (i.e., *p*_1_ << *p*_2_). Then, combining the samples from X and Y in a single cohort results in a non-unity OR estimate, a false-positive genotype-phenotype correlation due population structure. As a second example, consider two populations X and Y, where in both the OR≈2 (Figure 1-c). As in the previous example, suppose *p*_3_ is much larger in X than in Y. Let the exact same number of diseased and healthy subjects be sampled in X and in Y. Then, combining the samples from X and Y in a single cohort results in an OR estimate that is approximately unity, in spite of the OR in both the X and Y populations being approximately 2. This second example illustrates population structure concealing a genuine genotype-phenotype correlation. For both examples, we emphasize how the OR, and thus the genotype-phenotype correlation, is identical in the X and Y populations. Population structure led to the false-positive and false-negative calls solely by affecting sampling.

**Figure 2:**
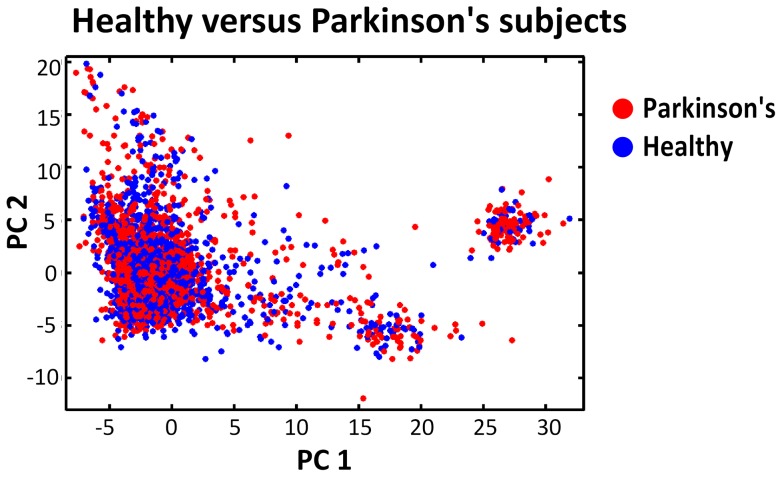
Distribution of patients and controls in the original cohort. The Hamza et al. [36] cohort healthy and Parkinson's individuals projected on the first 2 principal components of the SNP space. The cohort contains a total of 2000 Parkinson's patients and 1986 healthy controls.

**Figure 3:**
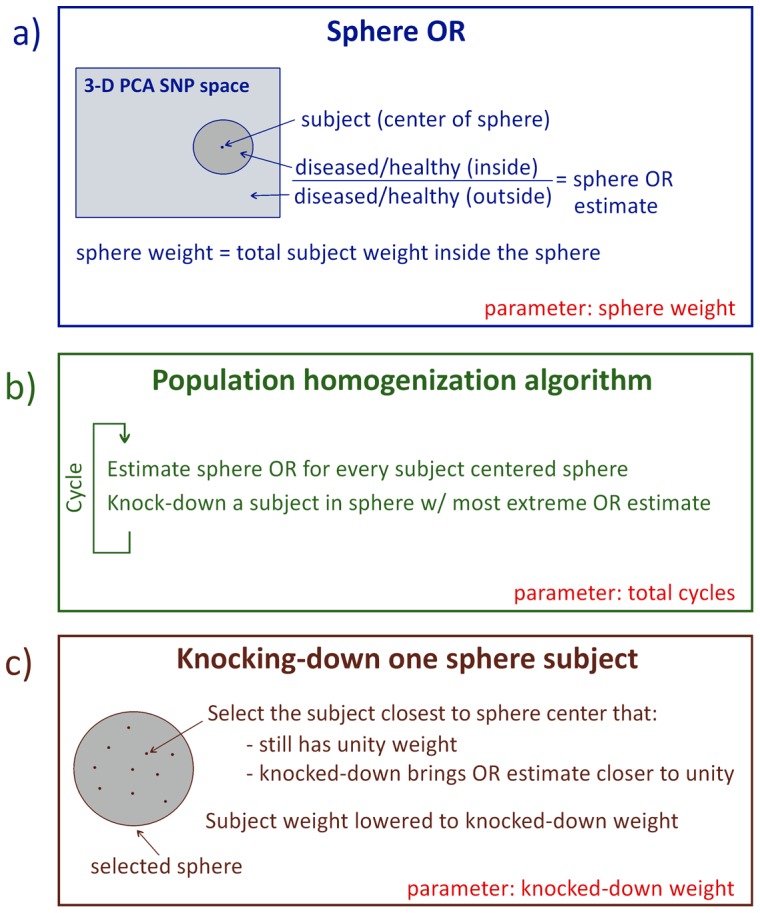
The population homogenization algorithm. **a**) Euclidean-distance spheres on the space defined by the first three principal component projections of the 75000 SNP space (3PC-space). The spheres are characterized by the total subject statistical weight in their interior, rather than by their spatial radius. The sphere OR is defined as the OR for an idealized genotype present in every subject in the interior of that sphere and in no subject outside of it. ORs are estimated for all spheres centered on a cohort subject and of a predefined sphere weight. **b**) Overview of the homogenization algorithm. **c**) Within the sphere with the most extreme OR estimate, one subject is selected to have its statistical weight knocked-down to a predefined knocked-down weight.

Transformations can be applied to the contingency table. Discarding diseased samples is akin to scaling by a less than unity common factor the contingency table entries *a* and *b* (Figure 1-a). Similarly, discarding healthy samples is akin to scaling by a less than unity common factor the entries *c* and *d*. Equivalently, we may always plausibly assume a cohort resulted from sampling a selected number of A subjects and a selected number of ∼A subjects, all under a given sampling partiality between diseased and healthy subjects. From this perspective, the three sampling DOFs could be expressed as:

**Figure 4:**
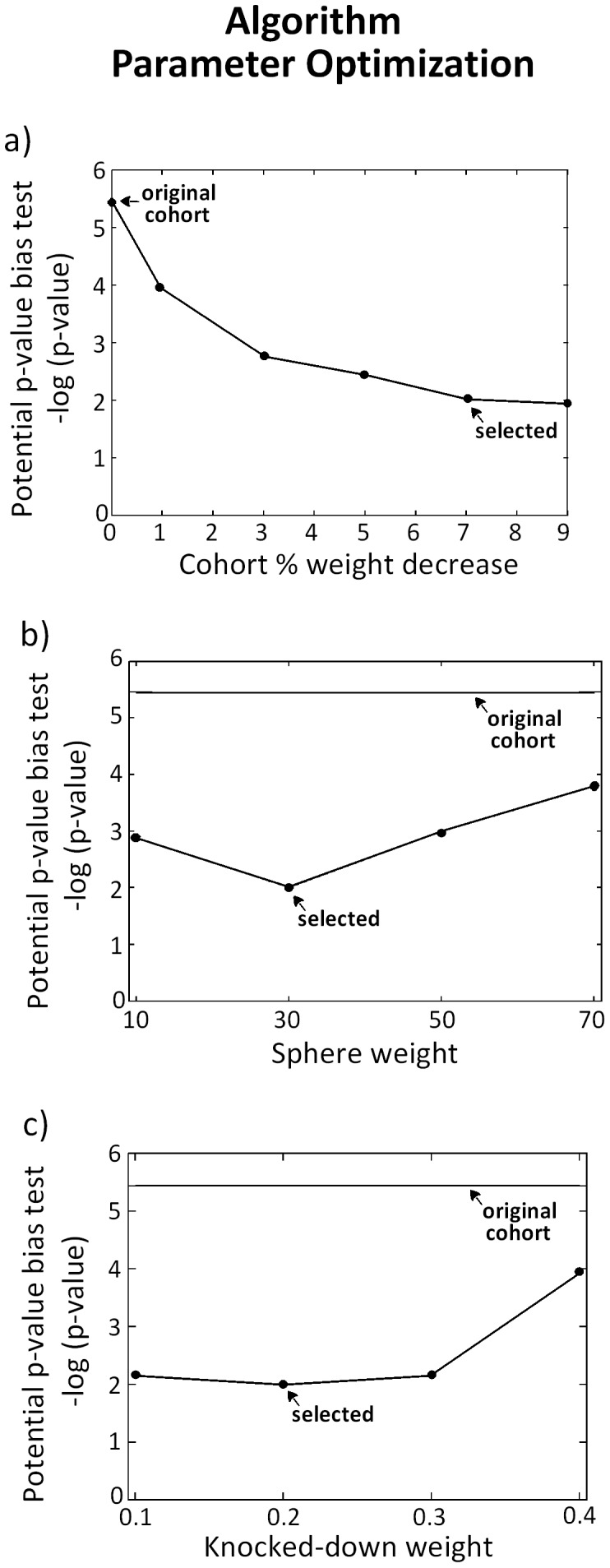
Algorithm parameter optimization. The algorithm was applied to homogenize the Hamza et al. cohort. The algorithm parameters were selected based on the value of the potential p-value bias test (see Table 1 and main text). Graph **a**) shows potential p-value bias versus % of cohort weight decrease. A 7% cohort weight decrease was selected, as weight decreases beyond this value produced only a marginal further decline in the potential p-value bias. **b**) The sphere weight parameter was set to 30. **c**) A 0.2 knocked-down weight was selected. In each of these graphs, the two parameters not represented are held at their selected values.

**Figure 5:**
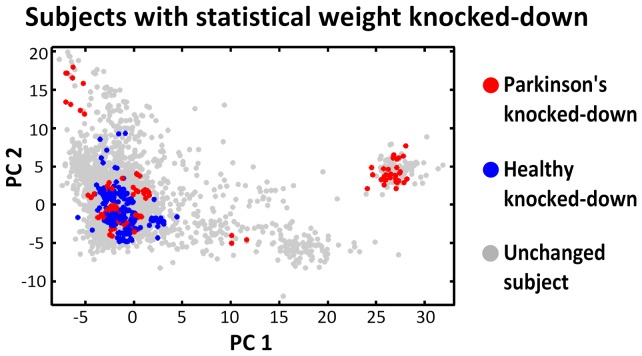
Statistically knocked-down individuals in the cohort homogenization process. The local homogenization of the Parkinson's/healthy marginal ratio (phenotype marginal matching) is performed by knocking-down the statistical weight of selected subjects. Overall, 120 of the 2000 Parkinson's patients and 230 of the 1986 healthy controls had their statistical weight knocked-down from 1 to 0.2. This represented a 7% net weight decrease in the cohort.

**Figure 6:**
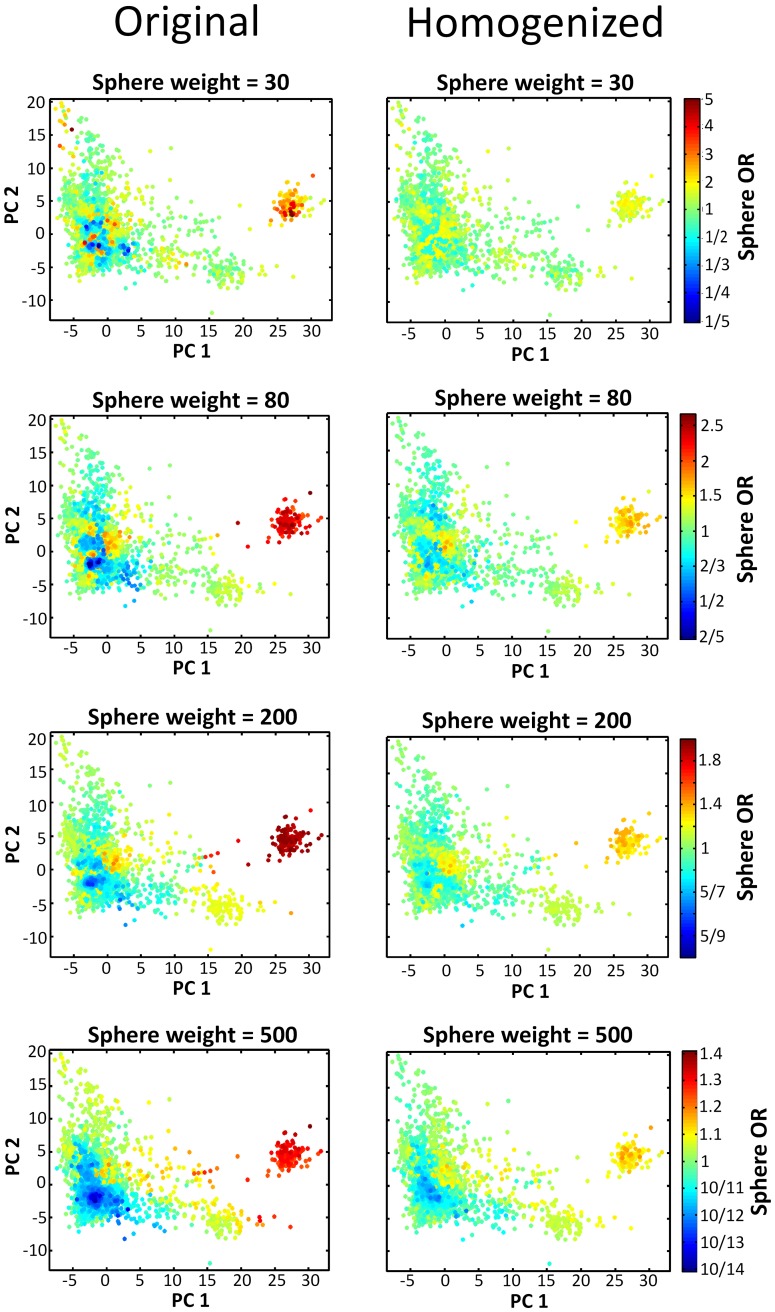
Estimated sphere ORs in the original and homogenized cohorts. Each subject is colored based on the estimated OR of a sphere centered on that subject and with the sphere weight stated on the top of the respective color map (see Figure 3-a for sphere OR definition). Sphere OR color scale reflects the symmetry of the OR definition. Note sphere OR color scale amplitude is smaller on color maps for larger sphere weights.

**Table 1:** The potential p-value bias test.

*Cohort weight*	Sphere weight	25	50	100	200	400	800	1600	*Potential p-value bias test*
*3986*	Original cohort	2*10^−4^	3*10^−4^	4*10^−6^	4*10^−6^	3*10^−4^	9*10^−5^	7*10^−5^	4*10^−6^
*3706*	Homogenized cohort	2*10^−2^	1*10^−2^	2*10^−2^	2*10^−2^	3*10^−2^	3*10^−2^	3*10^−2^	1*10^−2^

Consider the Fisher exact test p-value associated with an OR estimate for a hypothetical genotype present nowhere other than on every subject within a given spatial sphere. Each table entry reports the smallest such p-value, over all the subject-centered spatial spheres with the stated weight. These p-values provide a comparative measure of the potential for p-value bias due to population structure, before and after cohort homogenization. The value of the potential p-value bias test is the smallest of these p-values across all the tested sphere weights (rightmost column). Values shown pertain to application of the test to the original and homogenized (under the Figure 4 selected parameter values) Hamza et al. cohort. Note: for the homogenized cohort, the Fisher test is performed on the contingency table entries rounded to the nearest integer.

- *g*_1_ - number of A subjects sampled,
- *g*_2_ - number of ∼A subjects sampled and
- *g*_3_ - sampling partiality towards diseased subjects versus towards healthy subjects.

Now, discarding A samples is akin to scaling by a less than unity common factor the entries *a* and *c*. Similarly, discarding healthy samples is akin to scaling by a less than unity common factor the entries *b* and *d*.

**Figure 7:**
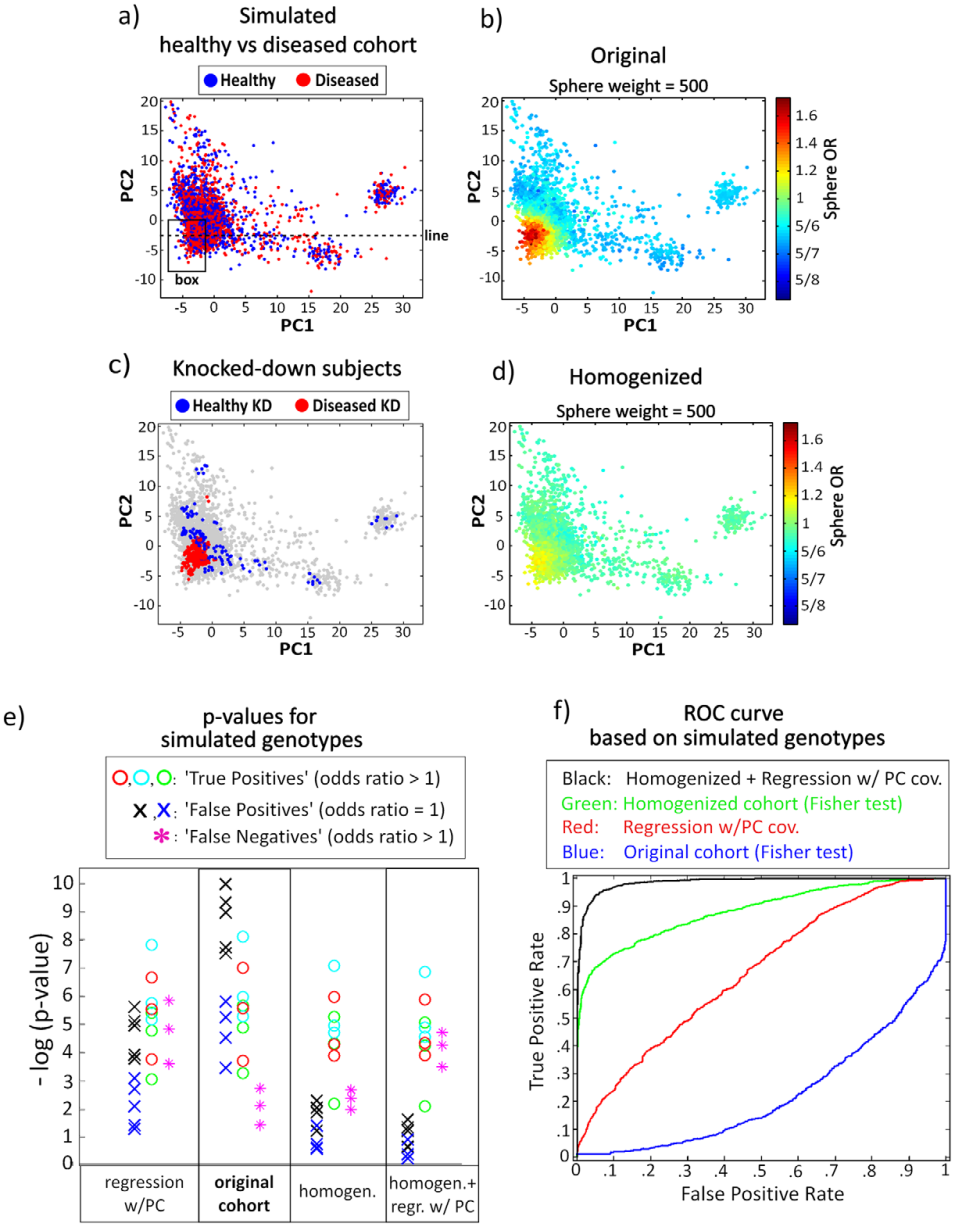
Simulation study. **a**) Synthetic diseased-healthy phenotype labels were assigned to the Hamza et al. cohort subjects. **b**) Visualization of the sphere ORs (at sphere weight = 500) in this synthetic dataset shows the imbalance in the diseased-healthy distribution. **c**) Individuals with their statistical weight knocked-down in the cohort homogenization process (final homogenized cohort weight reduction = 9%). **d**) Visualization of the sphere ORs (at sphere weight = 500), after the homogenization process. **e**) Synthetic genotype labels were assigned to individuals to produce the typical true-positive, false-positive and false-negative association (under no population structure correction). The -log(p-value) for these synthetic genotype-phenotype associations were calculated, based on four different approaches: Fisher exact test using the original cohort; Fisher exact test using the homogenized cohort; logistic regression with the 3 PCs as covariates; and logistic regression using the homogenized cohort weights and with the 3 PCs as covariates. **f**) Receiver Operating Characteristic (ROC) curves for the four different approaches. See Simulation Study Section for details.

Let us call *p*_3_ *matched cohorts* to those cohorts that could plausibly originate from a sampling of diseased and healthy subjects under the same partiality *p*_3_. Similarly, let us call *g*_3_ *matched cohorts* to those cohorts that could plausibly originate from a sampling of A and ∼A subjects under the same partiality *g*_3_. Only *p*_3_ or *g*_3_ matched cohorts are suitable to being merged. Fortunately, the abovementioned transformations can be utilized to produce matched cohorts. For instance, the transformations can always be used to equalize the corresponding marginal ratios in the two contingency tables (i.e., making the net A/∼A ratio equal on the two tables and making the net diseased/healthy ratio equal on the two tables). Doing so produces cohorts that are both *p*_3_ and *g*_3_ matched.

**Figure 8:**
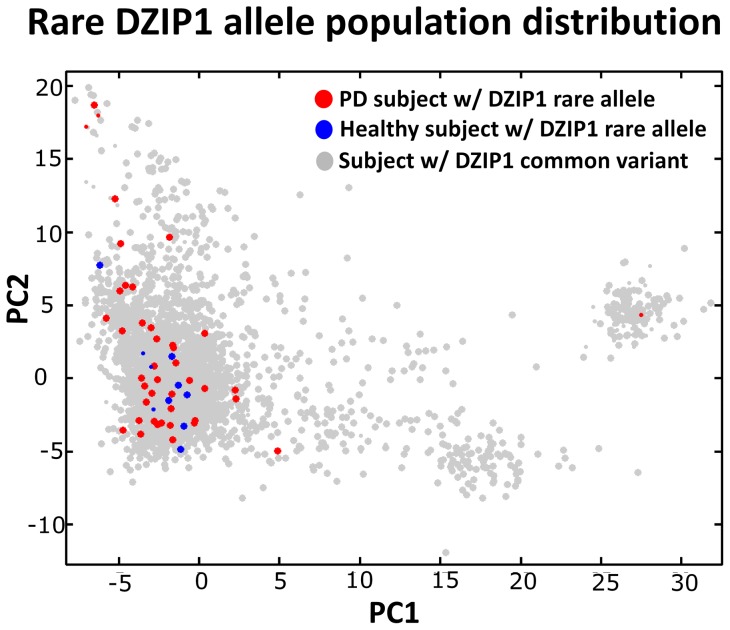
Interpreting the DZIP1 allele distribution in the context of population structure. Based on an analysis of the homogenized Hamza et al. cohort, a rare SNP mutation in the gene DZIP1 that increases the susceptibility to Parkinson's disease was found [39]. In this cohort, individuals carrying a copy of this rare DZIP1 allele are predominantly in a region where, as per the color maps of Figure 5, there is still a slight relative overabundance of healthy subjects (indicated by the blue hue in Figure 5). Thus, we conclude that the remaining population heterogeneity in the homogenized cohort has a propensity to diminish, rather than to inflate, the estimated OR of the DZIP1 SNP. Above, smaller dot sizes indicate subjects with knocked-down weight. Figure reproduced from Valente et al. [39].

In a GWAS, there is typically a vast number of genotype based population splits to consider and each requires its own cohort-matching transformation. Furthermore, population structure generally has a more complex continuous character than the two distinct populations assumed so far. Thus, computational-time wise cohort-matching for every genetic trait may not be feasible. Instead, we suggest equalizing the net diseased/healthy subject marginal ratio on the two cohorts. This can be achieved by appropriately discarding diseased or healthy samples (without regards to their genotype). Note that this equalization is independent of the genotype split under consideration. Now, if the OR is unity in the two populations, then the matching of the diseased/healthy subject ratio guarantees that the cohorts are *g*_3_ matched. This follows from an unity OR indicating that the diseased/healthy ratio in the population is identical for both genotypes under consideration. Note that the method is robust: in the limit where the individual population ORs approach unity and the cohort marginal diseased/healthy ratios approach each other, the cohorts approach being *g*_3_ matched. The limitation of the approach is that, for non-unity ORs, matching the diseased/healthy ratio no longer guarantees that the cohorts are *g*_3_ matched. This is clear in the Figure 1-c example, where the diseased/healthy ratio is unity in both cohorts and yet, the merged cohort produces a false-negative outcome. A careful examination of the example will show that the sampling is partial towards healthy subjects in population X relative to the sampling in population Y. Let us call *phenotype marginal matching* to this procedure of matching the diseased/healthy ratios in the cohorts. In summary, phenotype marginal matching reduces the false-positives (Figure 1-b case) but not the false-negatives (Figure 1-c case). However, the computational faster time, due to a single homogenization serving for all genotype pairs, makes it a practical choice. Finally, note that throughout we have assumed that the OR is the same in both populations. However, the OR could also be population dependent. As an example, consider a SNP that is a risk factor only under the net genetic background or dietary habits characteristic of a particular population. Such cases always benefit from an individualized analysis of the population cohorts, as their merging unavoidably produces some form of averaged OR estimate.

A commonly applied test to the cohort contingency table is the Fisher exact test [38]. Importantly, note that the test is valid on a contingency table from merged cohorts that were phenotype marginal matched, since the unity OR assumption of the test also guarantees that the cohorts are *g*_3_ matched.

### Continuous population structure case

So far we have assumed two well-defined, distinct populations. We now describe our phenotype marginal matching approach for the practical case where the population structure has a continuous character. The method is presented alongside its application to homogenization of the Hamza et al. Parkinson's GWAS cohort [36], [39]. This cohort of 2000 Parkinson's disease patients and 1986 controls was genotyped at on the order of 10^6^ SNPs. Subjects were recruited in North America and reported European ancestry. We first identified the population structure as Hamza et al. [36], via principal component analysis [16], [36]. The relative overall location of individuals in SNP space (Euclidean distance wise) reflects the cohort population structure. However, there are regions of the genome with a particular high density of genotyped, highly correlated SNPs. This results in a few such groups of SNPs overwhelmingly determining the clustering of subjects in SNP space. Following standard procedure [36], this was avoided by utilizing only a reduced set of 75 000 SNPs, selected for their comparatively lower correlation. The SNPs were selected using the program Plink [32], with a 50-SNP sliding window, shifting 5 SNPs with each move and recursively removing SNPs with r^2^>0.1. As discusssed in Hamza et al., the population structure is well captured by the first three principal components of this 75 000 SNP space. In here, our objective is therefore to homogenize the diseased/healthy subject ratio (phenotype marginal match) throughout the subspace defined by these first three principal components (henceforth 3PC-space). The uneven distribution of healthy and Parkinson's subjects in this space is visually perceptible in Figure 2 (third principal component not shown).

The homogenization is performed by lowering the statistical weight (‘statistical’ subsumed henceforth) of select subjects from their original unity weight, to a smaller, but nonzero, predefined *knocked-down weight*. Henceforth, let all spatial references be with respect to 3PC-space and based on Euclidean distances. We define the *sphere OR* as the OR for an idealized genotype present nowhere other than on every subject within that spatial sphere (Figure 3-a). Such a genotype thus marks exactly the population present within the sphere. The homogenization algorithm will employ spheres centered on cohort subjects. In lieu of sphere spatial radius, a more relevant parameter is *sphere cohort weight*, the total weight of the cohort subjects inside the sphere. This is due to the pertinent statistical comparison, as described below, being between spheres with the same cohort weight, not between spheres with the same spatial size per se.

We now describe the iterative homogenization algorithm (Figure 3-b,c). The sphere OR for every sphere with a predetermined cohort weight and centered around a cohort subject is estimated (note on weight discreteness: in practice, utilize the smallest sphere centered on the subject that equals or surpasses the desired weight). The sphere with the most extreme OR estimate is selected (given the symmetry in the OR definition, by extreme it is meant furthest from unity, in the sense whereby OR = 3 and OR = 1/3 are equally distanced from unity). Next, the weight of one cohort subject within this selected sphere is knocked-down in order to bring the sphere estimated OR closer to unity. The subject closest to the sphere center, still with unity weight and of the appropriate phenotype (diseased if the OR imbalance is due to too many diseased subjects within the sphere, healthy in the reverse case) is selected to have its weight knocked-down to the predefined knocked-down weight. The cycle is then repeated, with every sphere OR being re-calculated, a new sphere with the most extreme OR being highlighted and, within it, a new subject being selected for knock-down. This cycle is performed a determined number of times (set by the desired final cohort weight) upon which the procedure is concluded.

The algorithm requires selecting values for three parameters: total cycles (or final decrease in cohort weight), sphere weight and knocked-down weight (Figure 3). To select these, we introduce the *potential p-value bias test*. This is a measure of the OR estimate Fisher p-value bias potential due to population structure in the cohort. Consider again a hypothetical genotype present on every individual within a given spatial sphere and on none outside that sphere. This constitutes an extreme case of a genotype associated with a specific population (in this case, the population within the sphere). This genotype produces a false-positive OR estimate if the diseased/healthy ratio is not identical inside and outside the sphere. The smallest p-value that can be generated by such hypothetical genotypes thus provide a measure of the potential for p-value bias introduced by population structure. We numerically implement this test by considering all spheres centered on cohort subjects, hence effectively covering every relevant sphere location in 3PC-space. Similarly, a spectrum of sphere weights is considered. The value of the potential p-value bias test is then the smallest of the p-values thus generated.

The algorithm was applied to the Hamza et al. cohort. Based on the potential p-value bias test, the parameters were set to cohort weight decrease = 7%, sphere weight = 30 and knocked-down weight = 0.2 (Figure 4). We underline the limited sensitivity of the potential p-value bias test results to the selected parameter values, indicating the algorithm is robust in this regard (Figure 4). Table 1 breaks down the potential p-value bias test over the range of sphere weights tested. From the original Hamza et al. cohort to the homogenized cohort under the above selected parameters, the extreme p-value found over the spectrum of tested locations and weights decreased by four orders of magnitude, from 4*10^−6^ in the original cohort, to 1*10^−2^ in the homogenized cohort. Figure 5 shows the knocked-down individuals in the homogenization process. Figure 6 compares estimated sphere ORs on the original and homogenized cohorts. The comparison is performed across a range of sphere weights.

### Simulation study

Utilizing synthetic data, we compared the effect that homogenizing a cohort has on the GWAS typical true-positive, false-positive and false-negative association. We still relied on the real data Hamza et al cohort. However, the following synthetic disease/healthy distribution was now utilized (Figure 7-a): Diseased/healthy labels were assigned to individuals with a 0.5/0.5 chance in the population at large, except for individuals in the rectangular box region of Figure 7-a, where a 0.6 vs. 0.4 diseased vs. healthy chance was used when assigning the labels. In reality, this boxed region delimits approximately cohort individuals of Irish or English ancestry (based on self-reported ancestry, see Figure 2 in Valente et al. [39]). The created imbalance in the diseased/healthy distribution is apparent in Figure 7-b. The homogenizing algorithm was applied to this dataset (algorithm parameters: cohort weight decrease = 9%, sphere weight = 30 and knocked-down weight = 0.2). Figure 7-c shows the individuals knocked-down in the homogenization process. Comparison of Figure 7-d with Figure 7-b shows the effect of the homogenization procedure. Next, we created a number of synthetic genotypes to observe the effect of the homogenization procedure on the typical false-positive and false-negative association induced by population structure, as well as on the standard true-positive association (Figure 7-e).

To generate typically true-positive associations (under no population structure correction), genotype labels were assigned to all cohort individuals as follows:

1. For a diseased-phenotype individual there was a 0.57 probability of a ∼A genotype assignment, while for a healthy-phenotype individual this probability was 0.50 (yielding an OR = 1.32). The process was repeated five hundred times, thus generating five hundred such genotypes. The red circles in Figure 7-e correspond to three such genotypes.
2. For a diseased-phenotype individual there was a 0.15 probability of a ∼A genotype assignment, while for a healthy-phenotype individual this probability was 0.10 (yielding an OR = 1.59). The process was repeated five hundred times, thus generating five hundred such genotypes. The cyan circles in Figure 7-e correspond to three such genotypes.
3. For a diseased-phenotype individual there was a 0.06 probability of a ∼A genotype assignment, while for a healthy-phenotype individual this probability was 0.03 (yielding an OR = 2.06). The process was repeated five hunderd times, thus generating five hundred such genotypes. The green circles in Figure 7-e correspond to three such genotypes.

The above genotypes, spanning a range of minor allele frequencies, are homogeneously distributed across the population. Therefore, evaluation of their association with phenotype status is not distorted by population structure effects.

To generate typically false-positive associations (under no population structure correction), genotypes labels were assigned to all cohort individuals as follows:

1. For an individual inside the rectangular box (Figure 7-a), there was a 0.90 probability of a ∼A genotype assignment, regardless of phenotype status. For an individual outside the rectangular box, there was a 0.10 probability of a ∼A genotype assignment, regardless of phenotype status. The process was repeated five hunderd times, thus generating five hundred such genotypes. The black crosses in Figure 7-e correspond to four such genotypes.
2. For an individual inside the rectangular box, there was a 0.80 probability of a ∼A genotype assignment, regardless of phenotype status. For an individual outside the rectangular box, there was a 0.20 probability of a ∼A genotype assignment, regardless of phenotype status. The process was repeated five hunderd times, thus generating five hundred such genotypes. The blue crosses in Figure 7-e correspond to four such genotypes.

The above genotypes have no effect on the phenotype status (OR = 1). However, the ∼A genotype is highly common in the rectangular box population, by comparison with its presence in the population at large. In accordance with the Figure 1-b example, the concurrent higher prevalence of both the ∼A genotype and the diseased phenotype in the rectangular box population region generates a false apparent genotype-phenotype association.

To generate typically false-negative associations (under no population structure correction), genotypes labels were assigned to all cohort individuals as follows:

1. For an individual below the dashed line on Figure 7-a (PC2<−2.68) and a disease phenotype, there was a 0.08 probability of a ∼A genotype assignment. For an individual below the dashed line on Figure 7-a and a healthy phenotype, there was a 0.05 probability of a ∼A genotype assignment. For an individual above the dashed line on Figure 7-a and a disease phenotype, there was a 0.95 probability of a ∼A genotype assignment. For an individual above the dashed line on Figure 7-a and a healthy phenotype, there was a 0.92 probability of a ∼A genotype assignment. The process was repeated five hunderd times, thus generating five hundred such genotypes. The magenta stars in Figure 7-e correspond to three such genotypes. In reality, the dashed line roughly delimits from above individuals of Irish or Italian ancestry (based on self-reported ancestry, see Figure 2 in Valente et al. [39]).

For the above genotypes, OR = 1.65 in both the above and the below the dashed line populations. However, the ∼A genotype is highly prevalent in the population above the dashed line, while the A genotype is highly prevalent in the population below the dashed line. This is enough to conceal the genotype-phenotype association. Note that the diseased/healthy ratio is roughly identical in the above and below the dashed line populations. As previously illustrated by the Figure 1-c example, an homogeneous diseased/healthy phenotype ratio across the population does not prevent these false-negatives cases.

Figure 7-e shows -log(p-value) for some of these synthetic genotypes, based on four different approaches:

1. Original cohort: using the original cohort and the Fisher exact test.
2. Regression w/PCs: using the original cohort and logistic regression with the first 3 PCs as covariates to account for population structure effects.
3. Homogenized: using the homogenized cohort and the Fisher exact test.
4. Homogenized + regression w/PCs: using the homogenized cohort and logistic regression with the first 3 PCs as covariates (the knocked-down homogenized cohort weights are employed in the logistic regression).

The true-positives, false-positives and false-negatives behave as expected when not accounting for population structure. Both logistic regression and the cohort homogenization method are able to decrease -log(p-value) of the false-positives. However, the reduction is significantly more pronounced using the cohort homogenization method. The average -log(p-value) of true-positives is not significantly decreased by the 9% smaller size of the homogenized cohort. The decrease is comparable to the one observed under logistic regression, being in this latter case attributable to the addition of the 3 PCs as covariates. As expected, false-negatives are not rescued by the homogenization method. Their -log(p-value) does significantly increased under logistic regression. Finally, the overall best results were obtained by combining the homogenization and logistic regression methods: The -log(p-value) of false-positives was reduced the most, true-positives were again only marginally affected, and false-negatives were still rescued by the logistic regression.

Using the entire set of simulated genotypes, we built Receiver Operating Characteristic (ROC) curves for each of the above four approaches, showing the attainable combination of true positive and false positive rates, depending on the selected p-value significance level. The ROC curves confirm the observations based on the few genotypes individually analyzed in Figure 7-e, namely with the ROC curves being ordered from best to worst as 1) Homogenized + regression with PCs, 2) Homogenized, 3) Regression with PCs and 4) Original cohort.

## Results and Discussion

In this article we i) propose cohort homogenization as a strategy for minimizing false-positives in a GWAS, ii) present an algorithm for homogenizing a cohort and iii) introduce a measure for assessing p-value bias potential due to population structure. Note that although the homogenization method reduces false-positives, it is not able to guarantee a given false-positive rate. When applied to the Hamza et al. Parkinson's cohort, the method significantly reduced its p-value bias potential (Table 1). The intuitive character of the approach is also advantageous. For instance, in a separate work, this homogenized Parkinson's cohort was analyzed under the hypothesis-rich framework [39], [40]. The main finding was a rare SNP mutation in the gene DZIP1 that increases the susceptibility to Parkinson's disease. As shown in Figure 8, this rare DZIP1 mutant occurs predominantly in a region where, as per the color maps of Figure 6, there is still a slight relative overabundance of healthy subjects (marked by the blue end of the spectrum). Therefore it follows that the remaining population heterogeneity in the homogenized cohort has a propensity to diminish, rather than to inflate, the estimated OR of the DZIP1 SNP.

The homogenization approach introduced in this article opens a diversity of interesting research directions for future exploration. Firstly, the simulation work we performed highlights that the homogenization approach could potentially be profitably used in conjunction with other methods, namely logistic regression with the principal components as covariates. We are currently working on a thorough examination of such combined approaches. Secondly, our focus on homogenizing only the diseased/healthy marginal phenotype meant that false negatives induced by population structure are not addressed by the approach. However, they would be, if *both* phenotype and genotype marginals were homogenized. This is computationally demanding, due to requiring a new homogenization for every additional genotype pair being tested. However, perhaps variations on the approach or on the algorithm can make this full marginal matching computationally tractable and practical to apply to at least a considerable selection of genotype pairs. Thirdly, we have not shown optimality of the homogenization algorithm regarding the trade-off between achieved homogenization and imposed cohort weight reduction. Thus, the development of alternative more efficient homogenization algorithms is another open research problem.
